# Vitamin D Status Modulates Inflammatory Response in HIV+ Subjects: Evidence for Involvement of Autophagy and TG2 Expression in PBMC

**DOI:** 10.3390/ijms21207558

**Published:** 2020-10-13

**Authors:** Monica Currò, Giuseppa Visalli, Giovanni Francesco Pellicanò, Nadia Ferlazzo, Maria Giovanna Costanzo, Flavia D’Andrea, Daniela Caccamo, Giuseppe Nunnari, Riccardo Ientile

**Affiliations:** 1 Department of Biomedical and Dental Sciences and Morphofunctional Imaging, University of Messina, 98125 Messina, Italy; moncurro@unime.it (M.C.); gvisalli@unime.it (G.V.); nferlazzo@unime.it (N.F.); mgc90@hotmail.it (M.G.C.); dcaccamo@unime.it (D.C.); 2 Unit of Infectious Diseases, Department of Human Pathology of Adult and Childhood “Gaetano Barresi”, University of Messina, 98125 Messina, Italy; gpellicano@unime.it; 3 Unit of Infectious Diseases, Department of Clinical and Experimental Medicine, University of Messina, 98125 Messina, Italy; flavia.dandrea91@libero.it (F.D.); gnunnari@unime.it (G.N.)

**Keywords:** autophagy, cytokines, HIV, inflammation, peripheral blood mononuclear cells, tissue transglutaminase, vitamin D

## Abstract

Conflicting results on the involvement of vitamin D deficiency in inflammatory and immune response in HIV+ subjects are reported. We aimed to characterize the possible influence of vitamin D status on changes in expression of tissue transglutaminase gene (TGM2) and other genes involved in inflammatory response and autophagy in peripheral blood mononuclear cells (PBMC) from HIV+ subjects. HIV+ subjects (*n* = 57) under antiretroviral therapy (ART) and healthy controls (*n* = 40) were enrolled. mRNA levels of 1-alpha-hydroxylase (*CYP27B1*), tumor necrosis factor-α (*TNF-α*), interferon-γ (*IFN-γ*), *TGM2*, microtubule-associated protein 1A/1B-light chain 3 (*LC3*), autophagy-related 5 homolog (*ATG5*), and Beclin 1 (*BECN1*) were quantified by real-time PCR. In HIV+ subjects, 25(OH)D_3_ plasma levels were negatively correlated with time since HIV diagnosis. In PBMC from HIV+ subjects, increases in gene expression of *TNF-α* and *IFN-γ* in comparison to controls were observed. The highest increase in *TNF-α* transcripts was observed in HIV+ subjects with deficient 25(OH)D_3_ levels. Autophagy-related genes *LC3*, *ATG5*, and *BECN1* were down-regulated in HIV+ subjects. Moreover, *TGM2* transcripts were up-regulated in PBMC from HIV+ subjects with 25(OH)D3 deficiency. Changes observed in PBMC from HIV+ subjects appeared to be dependent on vitamin D status. The present results suggest that vitamin D deficiency is associated with changes in the expression of markers of inflammation and autophagy, resulting in immune cell dysfunction.

## 1. Introduction

Numerous clinical observations show that vitamin D deficiency may be associated with HIV infection [1,2,3]. Vitamin D status may also be related to side effects of antiretroviral therapy (ART) or other conditions associated with HIV infection [4,5,6,7,8,9]. An association between low vitamin D levels and increases in markers of inflammation including interleukin-6 (IL-6) and high-sensitivity C-reactive protein (hs-CRP) [10,11] has been reported, while higher vitamin D levels were independently associated with a lower risk of mortality and AIDS events [12].

The vitamin D active form, namely, 1,25(OH)_2_D_3_ is produced in the kidney and also in extrarenal sites, including immune cells, thanks to the presence of enzyme 1-alpha-hydroxylase (CYP27B1) [13,14]. Thus, vitamin D may be linked to autocrine and paracrine regulatory mechanisms, largely described in immune cells [13,14]. In recent years, the presence of receptors for 1,25-dihydroxyvitamin D3 [1,25(OH)_2_D_3_] has been recognized in many cell types, including peripheral blood mononuclear cells (PBMC), with a relevant role in inflammatory response. Consequently, the growing importance of the role of 1,25(OH)_2_D_3_ in both innate and adaptive immune responses has been highlighted [15].

Decreases in CD4+ T lymphocytes were shown as the most relevant change in monitoring pathological conditions associated with HIV [16,17]. Although programmed cell death through apoptosis has been the most commonly described mechanism for lymphocyte depletion, more recent results give evidence of the occurrence of dysfunctional autophagy, which can be associated with both innate and adaptive immunity. It has been shown that HIV infection, leading to autophagy down-regulation in peripheral blood mononuclear cells (PBMC), can be related to mechanisms that permit viral replication but also allow sufficient autophagy for cell survival [18,19,20].

Autophagy plays a critical role in the homeostasis of inflammatory cells, such as macrophages, neutrophils, and lymphocytes, which may also be modulated by non-classical effects of vitamin D [21]. It has been demonstrated that adequate concentrations of 1,25(OH)_2_D_3_, inhibit *Mycobacterium tuberculosis* and HIV replication in coinfected macrophages through autophagy activation. In particular, the involvement of autophagy has been demonstrated by inhibiting the genes coding for Beclin 1 (BECN1) and autophagy-related 5 homolog (ATG5), two proteins specifically required for autophagy [22].

A large amount of data have underlined the fact that inflammatory reactions are early events leading to cell responses by which HIV infection increases the risk of various pathological conditions. Tissue transglutaminase (TG2), an ubiquitous member of the transglutaminase enzyme family, has been proposed as an early marker of inflammatory response activation [23,24,25,26]. Particular emphasis has been given to the induction of *TGM2*, the gene coding for TG2, in immune system cells for monitoring disease progression in HIV-infected individuals [27]. Recently, in healthy subjects with vitamin D deficiency, we gave evidence of a concomitant increase in mRNA levels of *TGM2* and pro-inflammatory cytokines, as well as biomarkers of cell adhesion, such as intercellular adhesion molecule 1 (*ICAM)* and lymphocyte function-associated antigen 1 (*LFA-1)*, involved in immune activation, indicating that TG2 may be a marker of PBMC activation [24,28]. While there are many studies on the relationship between HIV infection and vitamin D status, there are few results to emphasize the relation between vitamin D and inflammatory response associated with HIV in PBMC. The purpose of our study was to characterize the possible influence of vitamin D status on changes in the expression of *TGM2* and other genes, involved in inflammatory response and autophagy, in PBMC from HIV+ subjects.

## 2. Results

A total of 57 HIV+ subjects (mean age: 43.2 ± 1.8; 34 males and 23 females) under ART for at least 1 year were enrolled in this study. The main characteristics are given in Table 1. In parallel, 40 healthy subjects that were age- and sex-matched (mean age: 41.5 ± 1.6; 24 males and 16 females) were included in the control cohort.

In HIV+ subjects, the plasma concentrations of 25-hydroxyvitamin D3 [25(OH)D_3_] were 59.9 ± 3.6 nmol/L, which were lower than the normal reference range (75–200 nmol/L) and significantly lower than those in healthy subjects (87.4 ± 4.9 nmol/L, *p* < 0.001).

According to previous studies [28,29,30], HIV+ subjects included in our study were divided into three groups as follows: 25(OH)D_3_ deficiency (≤50 nmol/L), insufficiency (51–74 nmol/L), and sufficiency (≥75 nmol/L).

In particular, among the HIV+ subjects, only 15 had sufficient 25(OH)D_3_ plasma levels (96.9 ± 3.7 nmol/L), while 17 subjects had insufficient (63.4 ± 1.3 nmol/L) and 25 subjects deficient (35.4 ± 1.8 nmol/L) 25(OH)D_3_ plasma levels. Instead, most of the controls (*n* = 25) had sufficient 25(OH)D_3_ levels (106.4 ± 4.2 nmol/L), while only nine and six healthy subjects had insufficient (65.8 ± 2 nmol/L) and deficient (40.6 ± 3.4 nmol/L) 25(OH)D_3_ levels, respectively.

**Figure 1:**
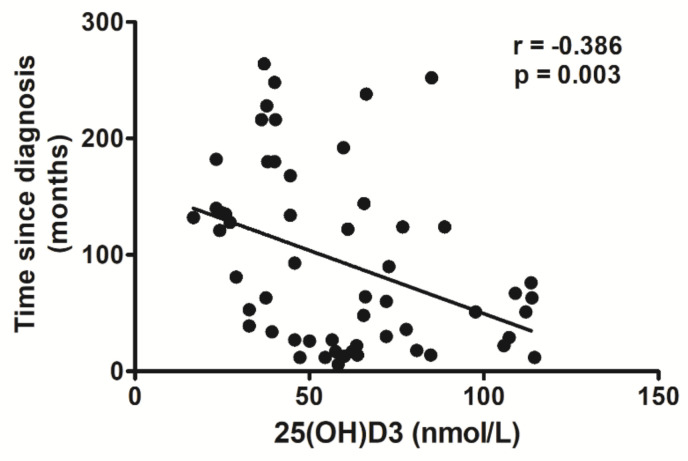
Correlation analysis between 25(OH)D_3_ plasma levels and time since HIV diagnosis.

As reported in Figure 1, the 25(OH)D_3_ depletion was associated with HIV infection. Indeed, plasma concentrations of 25(OH)D_3_ in HIV+ subjects were negatively correlated with time since HIV diagnosis (*r* = −0.386, *p* = 0.003).

Multiple linear regression analysis, including age as an independent variable, demonstrated that 25(OH)D_3_ concentrations were dependent on time elapsed from HIV diagnosis (standardized coefficient beta = −0.441, 95% CI: −0.761 to −0.121, *p* = 0.008), but independent of patient’s age (standardized coefficient beta = −0.09, 95% CI: −0.230 to 0.410, *p* = 0.576).

To ascertain possible changes in cell signaling associated with different vitamin D status in HIV+ subjects, we evaluated mRNA transcripts of genes involved in vitamin D activation, inflammation, and autophagy in PBMC from HIV+ subjects as well as healthy subjects.

**Figure 2:**
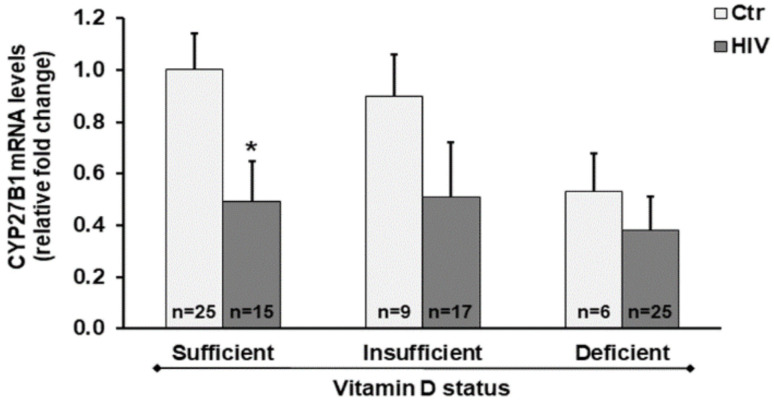
*CYP27B1* expression in peripheral blood mononuclear cells (PBMC) from HIV+ and healthy subjects. mRNA transcript levels were evaluated by real-time PCR. The results are expressed as mean ± SEM. * *p* < 0.05 significant difference in comparison to healthy subjects with sufficient 25(OH)D_3_ levels.

Notably, we found decreased *CYP27B1* mRNA levels in HIV+ subjects compared with healthy subjects. Even in HIV+ subjects with vitamin D sufficiency, we observed a significant downregulation of the CYP27B1 gene transcription in comparison to healthy subjects with vitamin D sufficient status (Figure 2).

**Figure 3:**
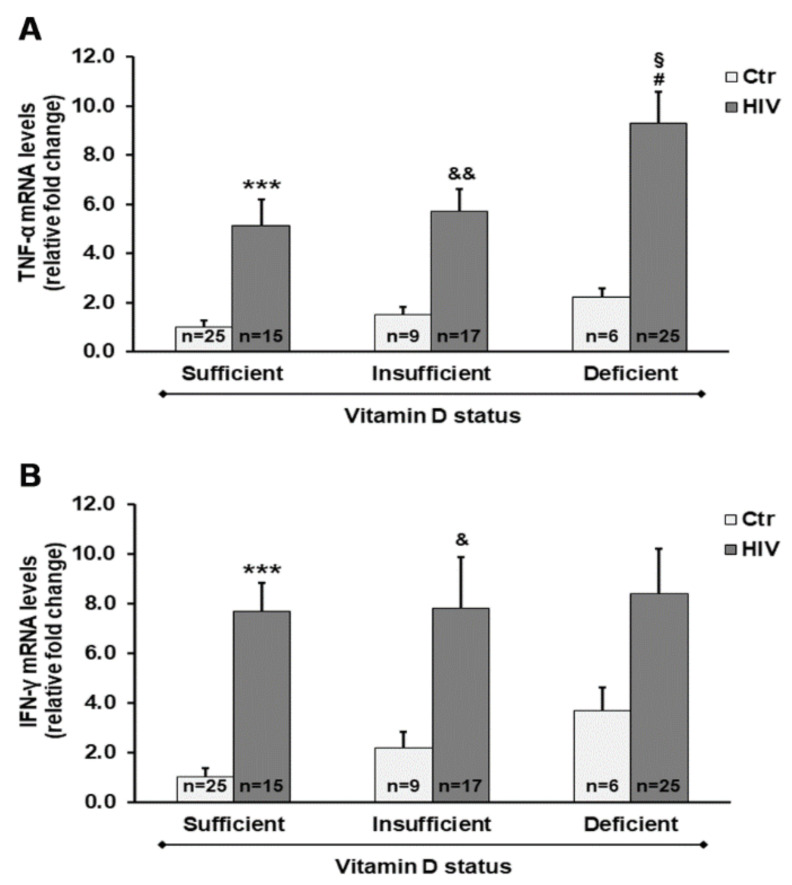
Gene expression of both tumor necrosis factor-α (*TNF-α*) (**A**) and interferon-γ (*IFN-γ*) (**B**) in PBMC from HIV+ and healthy subjects. mRNA transcript levels were evaluated by real-time PCR. The results are expressed as mean ± SEM. *** *p* < 0.001 significant differences in comparison to healthy subjects with sufficient 25(OH)D_3_ levels; ^&^ *p* < 0.05 and ^&&^ *p* < 0.01 significant differences in comparison to healthy subjects with insufficient 25(OH)D_3_ levels; # *p* < 0.05 significant differences in comparison to healthy subjects with deficient 25(OH)D_3_ levels; ^§^ *p* < 0.05 significant difference in comparison to HIV+ subjects with sufficient 25(OH)D_3_ levels.

As reported in Figure 3, we provide evidence for the influence of vitamin D status on changes in expression of pro-inflammatory mediators. In PBMC from HIV+ subjects, we observed a significant increase in gene expression of both tumor necrosis factor-α (*TNF-α*) and interferon-γ (*IFN-γ*) when compared with PBMC from control subjects. Of note, the most relevant increase in mRNA transcripts of *TNF-α* was observed in HIV+ subjects with deficient 25(OH)D_3_ levels.

We also assessed the mRNA transcript levels of different autophagy-related genes and *TGM2* in PBMC from HIV+ subjects with different 25(OH)D_3_ levels.

**Figure 4:**
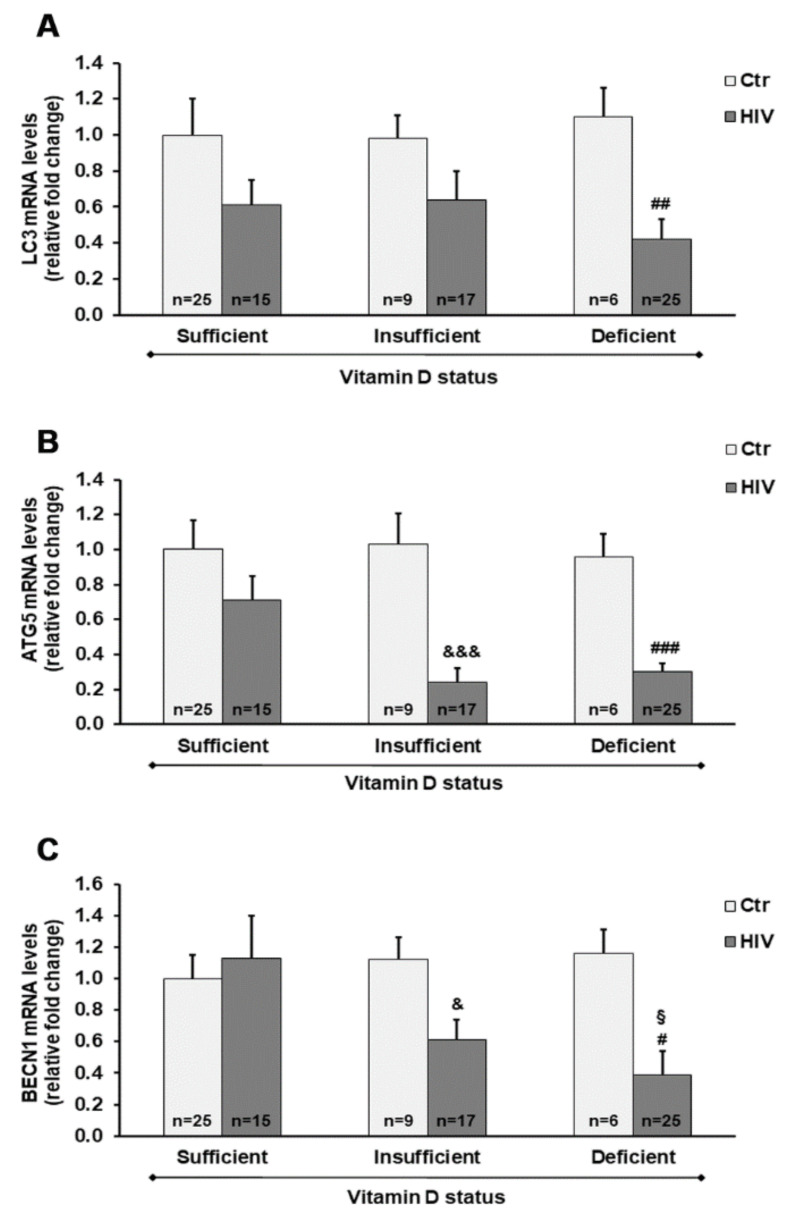
Gene expression of microtubule-associated protein 1A/1B-light chain 3 (*LC3*) (**A**), autophagy-related 5 homolog (*ATG5*) (**B**), and Beclin 1 (*BECN1*) (**C**) in PBMC from HIV+ and healthy subjects. mRNA transcript levels were evaluated by real-time PCR. The results are expressed as mean ± SEM. ^&^ *p* < 0.05 and ^&&&^ *p* < 0.001 significant differences in comparison to healthy subjects with insufficient 25(OH)D_3_ levels; # *p* < 0.05, ## *p* < 0.01, and ### *p* < 0.001 significant differences in comparison to healthy subjects with deficient 25(OH)D_3_ levels; ^§^ *p* < 0.05 significant difference in comparison to HIV+ subjects with sufficient 25(OH)D_3_ levels.

Overall, the microtubule-associated protein 1A/1B-light chain 3 (*LC3*), *ATG5*, and *BECN1* genes were downregulated in HIV+ subjects compared with healthy subjects. A highly significant downregulation of *ATG5* was observed in HIV+ subjects with either a deficient or insufficient vitamin D status, compared with controls. In addition, *LC3* and *BECN1* mRNA transcript levels were significantly reduced in HIV+ subjects with 25(OH)D_3_ deficiency, and were also reduced by about 40% (*p* > 0.05) in subjects with 25(OH)D_3_ insufficiency compared with healthy subjects (Figure 4).

**Figure 5:**
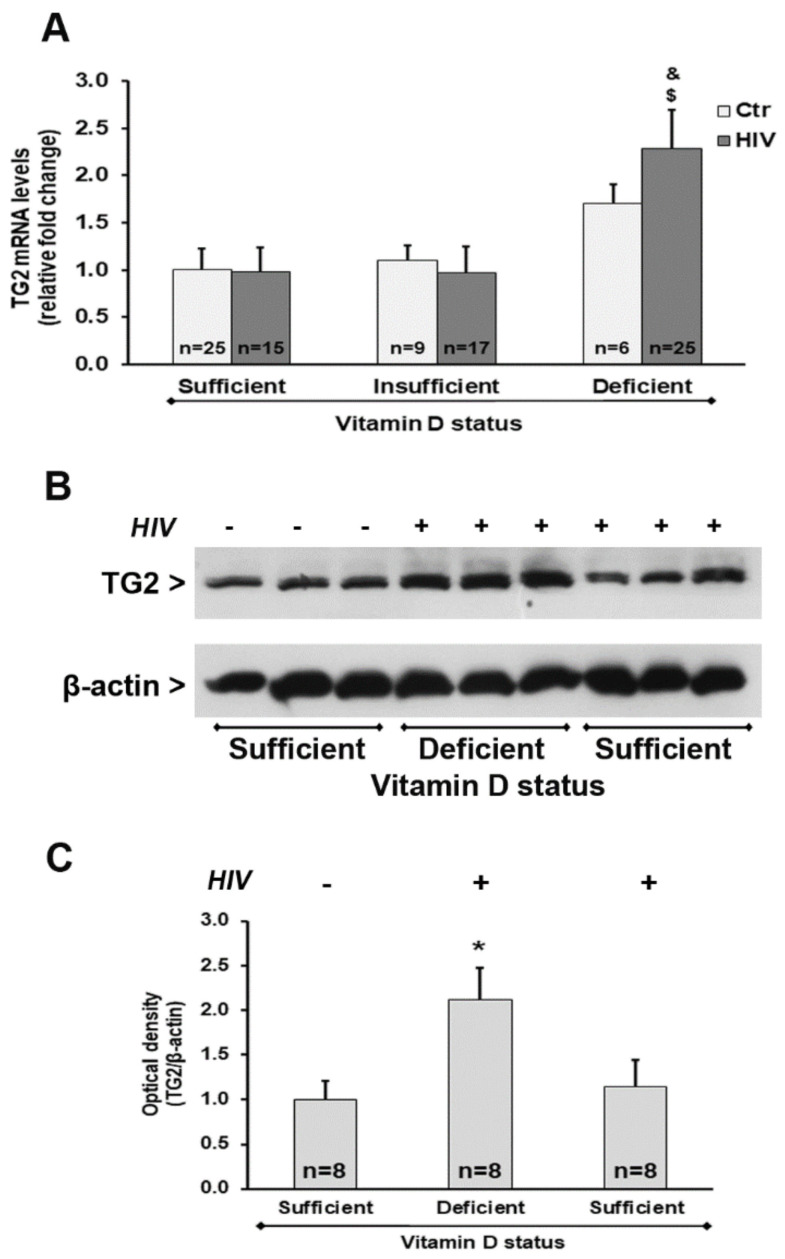
Tissue transglutaminase (TG2) expression in PBMC from HIV+ and healthy subjects. (**A**) mRNA transcript levels were evaluated by real-time PCR. The results are expressed as mean ± SEM. (**B**) Western blot analysis of TG2. (**C**) Densitometric analysis of immunoblots. The results are representative of the values (mean ± SEM) obtained from eight subjects for each group. * *p* < 0.05 significant difference in comparison to healthy subjects with sufficient 25(OH)D_3_ levels; ^$^ *p* < 0.05 significant difference in comparison to HIV+ subjects with sufficient 25(OH)D_3_ levels; ^&^ *p* < 0.05 significant difference in comparison to HIV+ subjects with insufficient 25(OH)D_3_ levels.

We also observed a significant up-regulation of *TGM2* mRNA transcript levels in HIV+ subjects with 25(OH)D_3_ deficiency compared with HIV+ subjects with either insufficient or sufficient levels of 25(OH)D_3_ (Figure 5A). Analysis of the protein expression confirmed these results, showing an increase of TG2 protein amounts in HIV+ subjects with 25(OH)D_3_ deficiency (Figure 5B,C).

Finally, as shown by the results of the generalized linear model (GLM), all genes (except *BECN1*) were significantly influenced by at least one of the predictors included in the model. In particular, expression of *TNF-α*, *IFN-γ*, and *ATG5* genes was dependent on HIV positivity and 25(OH)D_3_ levels. *CYP27B1* gene expression was significantly dependent on HIV infection, and a near significant dependence on 25(OH)D_3_ (*p* = 0.072) was also observed. *LC3* gene expression was only influenced by HIV infection, and *TGM2* was only influenced by 25(OH)D_3_ levels (Table 2).

## 3. Discussion

A decrease in vitamin D levels is frequently reported during HIV infection, and vitamin D supplementation is frequently used in the clinical treatment of HIV+ subjects. Besides numerous observations emphasizing the regulation of calcium/phosphate homeostasis, the involvement of vitamin D status in immune response is widely evident. Furthermore, HIV+ subjects with vitamin D receptor variants showed a significantly increased risk of progression to AIDS [31,32].

Present results also give evidence for an important relationship between vitamin D and disease progression. In our cohort of HIV+ subjects, we observed a negative correlation between 25(OH)D_3_ levels and time since HIV diagnosis. The low levels of 25(OH)D_3_ may be related to increased production of proinflammatory cytokines, such as TNF-α and IFN-γ, which in turn inhibit the effects of parathyroid hormone (PTH) and the hydroxylation of calcidiol [25(OH)D_3_] in the kidney, preventing the synthesis of active calcitriol [1,25(OH)_2_D_3_] [33,34]. Additionally, ART has been reported to influence vitamin D status in HIV+ subjects [35].

Further observations on different cell responses may be useful to define other approaches based on vitamin D supplementation for therapy and monitoring of HIV-associated pathological conditions. In this study, we found a decrease in mRNA transcript of *CYP27B1* gene in PBMC of HIV+ subjects. Thus, it is possible to hypothesize that the reduced expression of *CYP27B1* in PBMC could lead to low availability of 1,25(OH)_2_D_3_ in cells, promoting a proinflammatory phenotype. Changes in *CYP27B1* mRNA transcript levels potentially confirm the association between vitamin D deficiency and immune diseases. A previous study demonstrated that, in response to LPS stimulation, placentas from *CYP27B1* knockout mice expressed higher level of cytokines, chemokines, and chemokine receptors than *CYP27B1*^+/+^ placentas, highlighting the role for *CYP27B1* in the control of response to inflammatory stimuli [36]. Furthermore, in PBMC of healthy control subjects, the expression of the *CYP27B1* gene is dependent on a linkage disequilibrium block on chromosome 12, associated with several autoimmune diseases, as well as reduced expression of *CYP27B1* risk allele in tolerizing dendritic cells, which is consistent with reduced vitamin D function contributing to autoimmune disease risk [37]. Given that the *CYP27B1* enzyme exerts a critical role for cell specific functions, the potential to modulate 1,25(OH)_2_D_3_ production in specific tissues may improve the treatment of immune diseases [38].

In the present study, we also aimed to characterize the relationship between 25(OH)D_3_ plasma levels and inflammatory response of PBMC, which may be useful to study the underlying molecular mechanisms associated with endocrine, paracrine, and autocrine responses that are dependent on cholecalciferol metabolism and vitamin D status. We found that vitamin D levels affect the expression of *TNF-α* and *IFN-γ* in PBMC. These observations can largely be used to characterize the expression of relevant markers of cell modifications such as those responsible for inflammatory process.

An increase in the expression of cytokines, chemokines, and interleukins has been shown in HIV+ subjects, as well as in numerous inflammatory processes [39]. Increases of inflammatory mediators and activated monocyte phenotypes associated with vitamin D deficiency [40,41] have been related to tissue dysfunction, comorbidity development, AIDS progression, and death in HIV-infected people [42,43].

As recently reported, the mechanisms associated with HIV disease progression and the process of autophagy may play an essential role in acute and chronic inflammatory processes. Although the involvement of autophagy in different cell types from HIV+ subjects must be better clarified, recent evidence suggests that autophagy-mediated mechanisms may potentiate the recognition and degradation of the newly synthesized viral particles in immune infected cells. HIV infection leads to autophagy inhibition to prevent lysosomal degradation of HIV proteins, and thus several studies have indicated the potentially beneficial effects of pro-autophagy drugs to enhance the control of HIV infection [20]. On the basis of the present results, it is possible to suggest that vitamin D status can affect the expression of different autophagy markers. Indeed, *LC3*, *ATG5*, and *BECN1* were expressed at lower levels in PBMC from HIV+ subjects with 25(OH)D_3_ deficiency compared with HIV+ subjects with adequate 25(OH)D_3_ levels, and the *ATG5* gene expression was significantly dependent on 25(OH)D_3_ levels. The down-regulation of autophagy-related genes was associated with an increase of *TNF-α* and *IFN-γ* mRNA levels, also suggesting that autophagy may be involved in the control of cytokine production. As previously shown, several mechanisms underlying the modulatory effects of vitamin D on autophagy signaling have been demonstrated [44], i.e., vitamin D down-regulates expression and inhibits the activity of mammalian target of rapamycin (mTOR), a negative regulator of autophagy [45]. Moreover, cathelicidin, an essential protein in autophagosome formation, is a vitamin D receptor (VDR) target gene. Accordingly, vitamin D enhances Beclin 1 expression and autophagy through the up-regulation of cathelicidin [45].

We also first demonstrated that the decreased expression in biomarkers of autophagy was associated with elevated expression of *TGM2*, and that 25(OH)D_3_ levels represented a good predictor of *TGM2* transcription. TG2 is a well-known player in inflammatory conditions. During inflammation, monocytes are recruited into tissues and differentiated into macrophages [46]. Other results showed TG2 expression is highly up-regulated in monocytes during adhesion onto endothelial cells, indicating that TG2 is required for monocyte extravasation [47]. TG2 can mediate phagocytosis of apoptotic cells, a crucial process for resolution of inflammation and prevention of autoimmune disease development [25]. We previously reported the up-regulation of *TGM2* in PBMC in 25(OH)D_3_-deficient subjects. Furthermore, a significant positive correlation between *TGM2* expression and *TNF-α* mRNA levels was evident [24]. Similarly, the present results demonstrated a concurrent increase in *TGM2* and *TNF-α* mRNA levels, suggesting a relationship between expression of *TGM2* in PBMC and inflammatory response [24,28].

In the present study, however, the possible effects of vitamin D intake were not evaluated. Further studies are needed to determine whether vitamin D supplementation in HIV+ subjects induces changes in basal autophagy and immune response.

To summarize, the present results suggest that in HIV+ subjects, vitamin D deficiency may be associated with changes in expression of *TGM2* as well as other markers of inflammation and autophagy, resulting in immune cell dysfunction. The isolation of PBMC may be useful to characterize molecular mechanisms associated with inflammation and autophagy. However, the interaction of autophagy with pathological conditions such as HIV infection should be more deeply investigated.

## 4. Materials and Methods

### 4.1. Patient Recruitment

The study was conducted in accordance with the Declaration of Helsinki, and the protocol was approved by the local ethics committee (University of Messina, protocol number 10–20 28 January 2020). Informed written consent was obtained from all participants.

A total of 57 HIV+ subjects who attended the Unit of Infectious Diseases of the Polyclinic Hospital—University of Messina, were recruited for this study. All subjects were treated with ART, integrase strand transfer inhibitor plus 2 nucleoside reverse transcriptase inhibitors, according to recommendations on first-line antiretroviral regimens reported in the national guidelines of 2017, for at least 1 year. Thus, it was not possible to analyze therapeutic impact on examined markers of both inflammation and autophagy. Eligibility criteria required the absence of autoimmune disease, anti-inflammatory medications, and vitamin D supplementation.

The control group consisted of 40 healthy subjects matched for age and gender. Blood samples were collected in ethylenediaminetetraacetic acid (EDTA) tubes from both patients and controls. Plasma was obtained after blood centrifugation and stored at −20 °C until analysis.

### 4.2. Determination of 25(OH)D_3_ Plasma Levels

The quantitative determination of 25(OH)D_3_ plasma levels was performed by high-performance liquid chromatography (HPLC) with a Bio-Rad 25(OH)D_3_/D2 kit (Bio-Rad, Milan, Italy) according to the manufacturer’s instructions. Separation of 25-OH-vitamin D3 and internal standard took place on a reversed-phase cartridge followed by subsequent UV detection (λ = 265 nm) and quantitative evaluation.

### 4.3. PBMC Collection

PBMC were isolated by centrifugation on a Ficoll Histopaque density gradient. The blood collected in test tubes containing EDTA was diluted at a ratio of 1:2 in phosphate-buffered saline (PBS), layered on 4 mL of Ficoll, and centrifuged at 400× *g* for 20 min. PBMC, layered in the Ficoll plasma interface, were harvested, washed twice with PBS, and stored at −80 °C until use.

### 4.4. Quantitative Studies of Gene Expression

Total RNA isolation from PBMC was carried out using the TRIzol reagent (Invitrogen, Milan, Italy), according to the manufacturer’s instructions. Two micrograms of total RNA were reverse-transcribed into complementary DNA (cDNA) by using the High-Capacity cDNA Archive Kit (ThermoFisher Scientific, Milan, Italy). mRNA levels of *CYP27B1*, *TNF-α*, *IFN-γ*, *TGM2*, *LC3*, *ATG5*, and *BECN1* were quantified by SYBR green-based real-time PCR. The primer sequences used are listed in Table 3. Quantitative PCR reactions were carried out in 10 µL reactions containing 1× SYBR green PCR Mastermix, 0.1 µM specific primers, and 25 ng RNA converted into cDNA. β-Actin was used as endogenous control. Real-time PCR was performed in a 7900HT Fast Real-Time PCR System (Applied Biosystems, Foster City, CA, USA) with the following profile: one cycle at 95 °C for 10 min, followed by 40 cycles at 95 °C for 15 s and 60 °C for 1 min. For SYBR green assays, we added a standard dissociation stage to assess primer specificity. Data were collected with SDS 2.3 software (Applied Biosystems, Foster City, CA, USA) and analyzed using the 2 ^−ΔΔCt^ relative quantification method.

### 4.5. Power and Sample Size Calculation

A power analysis (ANOVA with fixed effects) was performed to establish the adequate number of subjects to be enrolled considering expression of *TNF-α* gene as primary outcome. Assuming an effect size of 0.4, an alpha error probability equal to 0.05, a power level of 0.8, and six groups, we found that a number of 90 subjects was necessary to ensure an adequate power. A posteriori, on the basis of different sample sizes within each group, we assessed that the achieved power was equal to 0.85. The software used for power analysis was G*Power, version 3.1.9.4.

### 4.6. Statistical Analysis

All values are expressed as mean ± standard error of the mean (SEM). Since all examined variables were normally distributed, as verified by the Kolmogorov–Smirnov test, we performed analyses by parametric tests. Comparisons between two groups were performed by Student’s *t*-test. Comparisons between more groups were carried out using one-way ANOVA followed by Bonferroni’s post-hoc test. To evaluate the relationship between two variables, we applied Pearson’s correlation analysis. The multiple linear regression test was used to assess the dependence of 25(OH)D_3_ plasma concentrations on time since diagnosis and age. Generalized linear models (GLM) were estimated for the expression of each gene in order to account for the influence of HIV infection and vitamin D levels, inserting potential confounders such as age; gender; and, only for HIV+ subjects, time since HIV diagnosis. Statistical analyses were performed using SPSS v22. Differences were considered significant for values of *p* < 0.05.

## Figures and Tables

**Table 1:** Main clinical characteristics of the 57 HIV+ subjects included in the cohort.

Characteristic	Mean ± SEM
Age	43.2 ± 1.8
Sex (female/male)	23/34
Time since HIV diagnosis (months)	93.1 ± 10.1
HIV viral load (copies/mL)	41.3 ± 26.4

SEM: standard error of the mean.

**Table 2:** Generalized linear model (GLM) for gene expression, accounting for the influence of HIV infection, 25(OH)D_3_ levels, gender, age, and time since HIV diagnosis.

	*CYP27B1* Gene Expression		
**Variables**	**B**	**95% C.I.**	***p*** **-Value**
HIV infection	−0.301	−0.4932; −0.110	**0.002**
Gender	0.116	−0.022; 0.254	0.098
Age	0.001	−0.007; 0.006	0.899
25(OH)D3 levels	0.002	0.000; 0.005	0.072
Time since HIV diagnosis #	0.001	−0.001; 0.002	0.283
	***TNF-α* gene expression**		
HIV infection	0.622	0.403; 0.842	**0.000**
Gender	0.075	−0.084; 0.233	0.355
Age	0.004	−0.003; 0.011	0.279
25(OH)D3 levels	−0.003	−0.006; −0.001	**0.022**
Time since HIV diagnosis #	0.001	−0.001; 0.002	0.546
	***IFN-γ* gene expression**		
HIV infection	0.606	0.313; 0.900	**0.000**
Gender	0.040	−0.171; 0.252	0.709
Age	−0.006	−0.016; 0.003	0.210
25(OH)D3 levels	−0.005	−0.008; −0.001	**0.018**
Time since HIV diagnosis #	−0.002	−0.005; 0.002	0.983
	***LC3* gene expression**		
HIV infection	−0.280	−0.499; −0.060	**0.012**
Gender	−0.024	−0.182; 0.134	0.763
Age	0.001	−0.006; 0.008	0.745
25(OH)D3 levels	0.002	−0.001; 0.005	0.176
Time since HIV diagnosis #	0.001	−0.001; 0.002	0.477
	***ATG5* gene expression**		
HIV infection	−0.334	−0.525; −0.143	**0.001**
Gender	0.086	−0.051; 0.224	0.218
Age	0.005	−0.001; 0.011	0.105
25(OH)D3 levels	0.003	0.001; 0.005	**0.019**
Time since HIV diagnosis #	−0.001	−0.003; 0.001	0.729
	***BECN1* gene expression**		
HIV infection	−0.168	−0.451; 0.0115	0.245
Gender	−0.092	−0.296; 0.113	0.379
Age	0.006	−0.003; 0.016	0.178
25(OH)D3 levels	0.002	−0.001; 0.006	0.263
Time since HIV diagnosis #	−0.001	−0.002; 0.002	0.874
	***TGM2* gene expression**		
HIV infection	0.025	−0.158; 0.209	0.787
Gender	−0.007	−0.139; 0.125	0.916
Age	0.002	−0.004; 0.008	0.490
25(OH)D3 levels	−0.003	−0.006; −0.001	**0.005**
Time since HIV diagnosis #	−0.002	−0.003; 0.001	0.925

Bold font for *p*-values indicates statistically significant differences at a level ≤ 0.05. # This variable has only been tested on HIV+ subjects.

**Table 3:** Primer sequences used for SYBR green real-time PCR.

Gene	Primer	Sequence 5′→ 3′
*β-ACT*	forward	TGGTTACAGGAAGTCCCTTGCC
*β-ACT*	reverse	ATGCTATCACCTCCCCTGTGTG
*CYP27B1*	forward	GGAACCCTGAACAACGTAGTC
*CYP27B1*	reverse	AGTCCGAACTTGTAAAATTCCCC
*TGM2*	forward	CCTTACGGAGTCCAACCTCA
*TGM2*	reverse	CCGTCTTCTGCTCCTCAGTC
*TNF-α*	forward	GTGAGGAGGACGAACATC
*TNF-α*	reverse	GAGCCAGAAGAGGTTGAG
*IFN-γ*	forward	GCAGCCAACCTAAGCAAGAT
*IFN-γ*	reverse	TCACCTGACACATTCAAGTTCTG
*LC3*	forward	CGGTGATAATAGAACGATACAAG
*LC3*	reverse	CTGAGATTGGTGTGGAGAC
*ATG5*	forward	TGCCTGAACAGAATCATCCTT
*ATG5*	reverse	CCAGCCCAGTTGCCTTAT
*BECN1*	forward	ACAGTGAACAGTTACAGATGGA
*BECN1*	reverse	CTCAGCCTGGACCTTCTC

Abbreviations: *β-ACT*, β-actin; *CYP27B1*, 1α-hydroxylase; *TGM2*, tissue transglutaminase; *TNF-α*, tumor necrosis factor-α; *IFN-γ*, interferon-γ; *LC3*, microtubule-associated protein 1A/1B-light chain 3; *ATG5*, autophagy-related 5; *BECN1*, Beclin 1.
